# Use of a counterfactual approach to evaluate the effect of area closures on fishing location in a tropical tuna fishery

**DOI:** 10.1371/journal.pone.0174758

**Published:** 2017-03-29

**Authors:** Tim K. Davies, Chris C. Mees, E. J. Milner-Gulland

**Affiliations:** 1 Department of Biology, Imperial College London, London, United Kingdom; 2 MRAG Ltd, London, United Kingdom; 3 Department of Zoology, University of Oxford, Oxford, United Kingdom; Havforskningsinstituttet, NORWAY

## Abstract

Spatial closures are widely used in marine conservation and fisheries management and it is important to understand their contribution to achieving management objectives. Many previous evaluations of closed area effects have used before-after comparisons, which, without controlling for a full range of factors, cannot ascribe changes in fleet behaviour to area closures *per se*. In this study we used a counterfactual approach to disentangle the effect of two closed areas on fishing location from other competing effects on the behaviour of the Indian Ocean tuna purse seine fishery. Our results revealed an inconsistent effect of the one of the closed areas between years, after taking into account the influence of environmental conditions on fleet behaviour. This suggests that the policy of closing the area *per se* was not the main driver for the fleet allocating its effort elsewhere. We also showed a marked difference in effect between the two closed areas resulting from their different locations in the fishery area. These findings highlight the need to account for other key fleet behavioural drivers when predicting or evaluating the contribution of area closures to achieving conservation and fishery management objectives.

## Introduction

The use of area closures is commonplace in addressing problems of sustainability in fisheries management [1–3]. Closures may be used for a variety of purposes, including safeguarding sensitive habitats, protecting vulnerable species or reducing fishing mortality on a fish stock. As with any management tool their performance in achieving these management objectives must be evaluated.

In the management of industrial tuna fisheries, area closures are most commonly used to reduce fishing mortality, either overall or for a particular component of the stock such as small or juvenile fish [4,5]. Short-term closures are typically placed in areas where catches or effort are historically high in order to restrict the access of fleets to the resource. As with other management actions, managers generally evaluate the efficacy of these area closures by measuring their impact on an indicator such as total catch or effort, for instance using before-after approaches [6–9]. The scale and intensity of observed change in the indicator is used to draw conclusions on the effectiveness of the closed area.

This approach is helpful in acknowledging change but does not reveal the full picture of how a management action has performed. For instance, while a before-after analysis can identify whether a change in total catch has occurred, it cannot ascribe changes in catch to the use of an area closure *per se*. Policy change is only one of many factors that can cause or contribute to an observed management impact. Environment, economic and social forces also influence how fishing fleets behave. In offshore tuna fisheries, the physical environment is particularly important in determining the movement of target tuna in space and time, and safety and cost considerations play an important role in determining a fisher’s decision to visit a particular area. These forces, which are usually independent of any management action, may disrupt the intended effect of management. Thus, without accounting for these different effects and isolating the effect of policy, managers risk misinterpreting the relationship between an action and an outcome.

A promising approach for diagnosing the reasons behind an observed change in a management indicator is to build up a counterfactual scenario. Counterfactual thinking considers what outcomes would have looked like in the absence of an intervention, yet while it is a common feature in some areas of policy impact evaluation (e.g. EU Better Regulation; http://ec.europa.eu/smart-regulation/guidelines/toc_guide_en.htm; accessed 27^th^ February 2017) it has not been widely adopted for evaluation of fishery management interventions. Here, a counterfactual approach can be achieved by developing a predictive model of fishing effort allocation that accounts for a range of influences on fleet movement. By comparing the observed response of a fleet to a closure to predictions of how the fleet would have behaved if the closure had not been implemented it is possible to isolate the effect of closures on fishery dynamics. An interesting use of the counterfactual approach in a manner broadly similar to this is demonstrated by [10], who revealed a negative effect of two marine reserves on the catch of a Gulf of Mexico reef fish fishery but, crucially, only after accounting for a range of behavioural and ecological factors.

In this study we used a counterfactual approach to isolate the effects on fishing behaviour of two closed areas in the western Indian Ocean: a temporary (and now discontinued) one-month fishery management closure off the Somalia coast, and the British Indian Ocean Territory (BIOT) marine reserve. We aimed to understand whether the policy of closing these areas *per se* caused a tuna purse seine fishing fleet to reallocate fishing effort, accounting for other key drivers of fleet behaviour.

Understanding the response of the purse seine fleet to the two area closures selected in this study is of interest for different reasons. In the case of the fishery closure, which was intended to temporarily reduce the fishing capacity of the purse seine fleet, it has been suggested that vessels may have reallocated effort in such a way as to maintain catches and therefore subvert the management outcome [11]. In the case of the BIOT marine reserve, some authors have expressed concern that the closure might displace purse seine fishing effort into areas of the Indian Ocean where the fishery is characterised by relatively high catch rates of small tunas and sharks, which may be more vulnerable to overfishing [12,13]. To begin to investigate these potential sustainability concerns in detail it is first necessary to examine whether the area closures themselves have had an impact on the spatial behaviour of the purse seine fleet.

## Materials and methods

### Closed areas

The two closures examined differed in their size, location and intended management objectives. In November 2011 and 2012 the Indian Ocean Tuna Commission (IOTC) designated a one-month closure that extended from the Somali coast to 60°E and covered a large part of the productive northwest fishing grounds typically fished by the fleet during August-November. This region is a key fishing area for the purse seine fleet and is characterised by consistently very high catches, mainly around floating objects [14]. The objective of the temporary closure was therefore to restrict the fishing capacity of the fleet and reduce fishing pressure, particularly on small and juvenile yellowfin and bigeye tunas (Resolution 10/01; http://www.iotc.org/English/resolutions.php; accessed 1st June 2013).

In late 2010 the British government designated their entire British Indian Ocean Territory a marine reserve. The BIOT reserve is positioned at the eastern periphery of the purse seine fishery area in a region typically fished during November-February and characterised by catches of free-swimming schools. Prior to the closure of BIOT, the importance of the area in terms of purse seine fishery production varied considerably from year to year, with the average proportion of total monthly catch taken within the Territory ranging from 0 to 23% (MRAG Ltd; unpublished data, 1999–2008). The designation of the BIOT reserve was not linked to regional fisheries management policy and whilst no formal management plan has yet been developed, several objectives of the reserve can be inferred, including the provision of a scientific reference site and near shore and pelagic biodiversity conservation [15].

### Statistical model

For the IOTC closure we focussed on the behaviour of the fleet during November, the month of the closure, in 2011 and 2012. For the BIOT reserve we focussed on the period December-January when the provision of fishing licences was historically highest (MRAG Ltd; personal communication). Fishing data were available until December 2012, allowing the analysis of two full fishing seasons. In both instances we chose to focus on the short term response of the fleet to the closures. Firstly, in a highly dynamic environment like the Indian Ocean it is difficult to accurately predict fleet behaviour in the long term (e.g. over a period of years), and any longer term effect of the closures on fishing location may be masked by other influences on fleet behaviour. Secondly, an understanding of the short term response of resource users is important for management decision making, which tends to operate over relatively short timeframes (e.g. annual cycles of scientific advice and policy making).

We used the generalised additive model (GAM) described in [16] to generate predictions of how the fleet would have allocated effort in the absence of the closures. We refer to these predictions as the ‘counterfactual’ scenario, and the true observations as the ‘observed’ scenario. The model was used to predict retrospectively the probability of effort being observed a location based on three main drivers acting on the behaviour of the fleet: 1) the response of skippers to the bio-physical conditions of the ocean; 2) practical constraints on movement of vessels; and 3) behavioural inertia in the use of certain fishing grounds at particular times of the year. Further description of this model is provided in the Supporting Information. To train the model, monthly fishing data were disaggregated by flag nationality and kept at their original spatial resolution of 1° latitude/longitude. Data were split into the periods 2007–2010 for model training and 2010–2012 for prediction.

## Results

### IOTC closure

**Fig 1:**
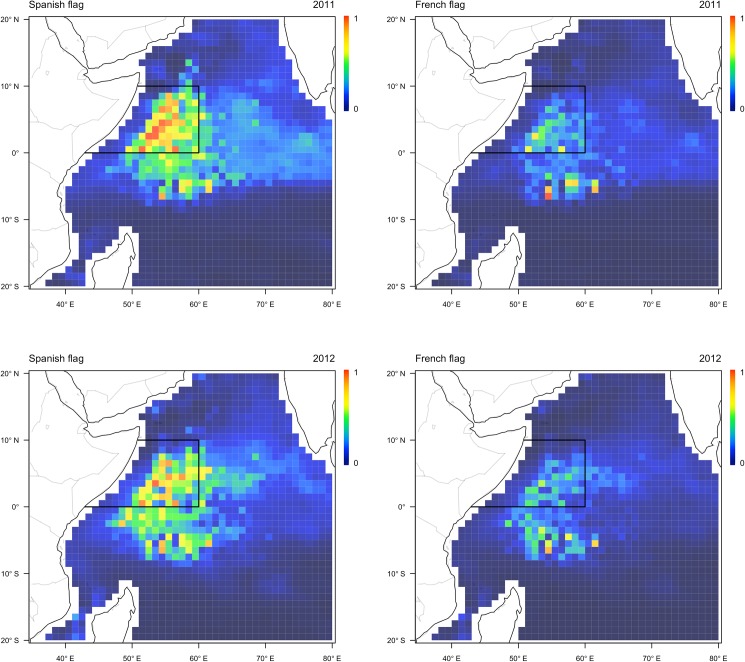
Predicted allocation of effort by the Spanish (left) and French (right) components of the fleet in the western Indian Ocean during November 2011 (upper) and 2012 (lower). The location of the IOTC area closure is shown by the solid line. Coloured cells show model predictions of probability of effort being allocated into an area.

In the absence of the closure the fleet was expected to have fished within the IOTC closure area in both years of its implementation. In this counterfactual scenario the predicted distribution of effort was noticeably different for the Spanish and French components of the fleet. The Spanish fleet component was expected to have fished across a large area extending from the Seychelles plateau in the south to the Omani coast in the north, with core fishing grounds predicted within the area of the IOTC closure. In comparison, the French fleet component was expected to have fished across a comparatively small area in subequatorial waters, with core grounds predicted on the Seychelles plateau to the south of the closure boundary (Fig 1). This difference in the predicted allocation of effort can be attributed to variation in fishing strategy, with Spanish vessels tending to target schools associated with floating objects at higher latitudes during the boreal summer months and French vessels moving into subequatorial grounds earlier in the year in pursuit of free swimming schools [16].

**Fig 2:**
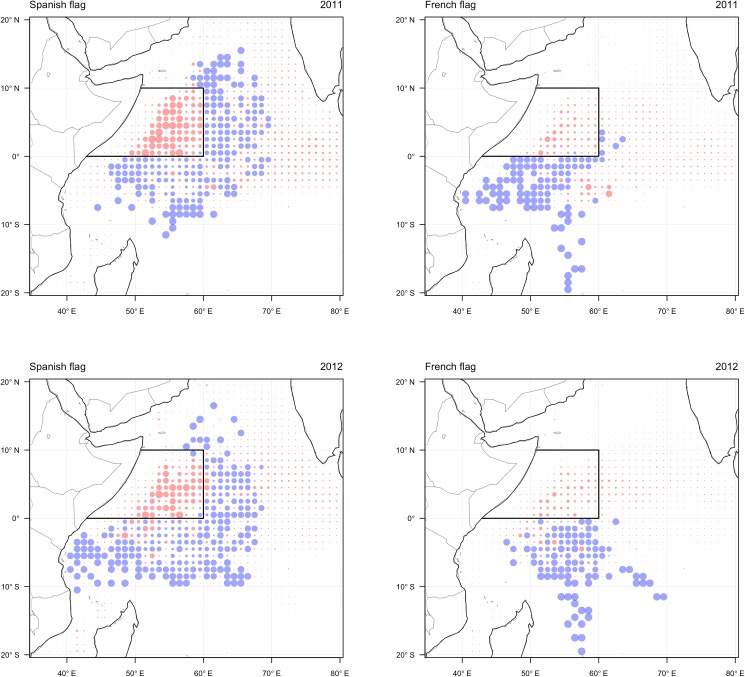
Observed allocation by the Spanish (left) and French (right) components of the fleet in the western Indian Ocean during November 2011 (upper) and 2012 (lower). Observations in each grid cell are shown as the predicted probability of effort minus observed response, where blue circles show more effort than expected and red circles show less effort than expected. Circle size indicates the relative size of the residual. The location of the IOTC area closure is shown by the solid line.

In the observed scenario the fleet complied with the closure in both years of its implementation and fishing effort was allocated to the south and east of the closed area boundaries (Fig 2). There was some similarity in the response of the Spanish and French fleet components, with both nationalities allocating the majority of fishing effort to the south of the closure. This behaviour largely corresponded with counterfactual predictions of effort allocation in the absence of the closure, suggesting that vessels mainly fished in the parts of their seasonal fishing grounds that remained accessible rather than moving to new grounds. The major difference in the response of the two flag nationalities was the allocation of effort by Spanish vessels to the east of the closure area, well beyond the eastern extent of the typical seasonal fishing grounds. This allocation of effort is probably explained by skippers searching for floating objects that had drifted eastwards out of the closure area, which would not be considered usual behaviour under typical conditions due to the reduced chance of finding and catching associated schools in this region (skipper; personal communication). This suggests that either fishing opportunities were not satisfactory in the accessible parts of the typical fishing grounds or that skippers were exploring eastern areas in an attempt to test out new fishing opportunities.

### BIOT reserve

**Fig 3:**
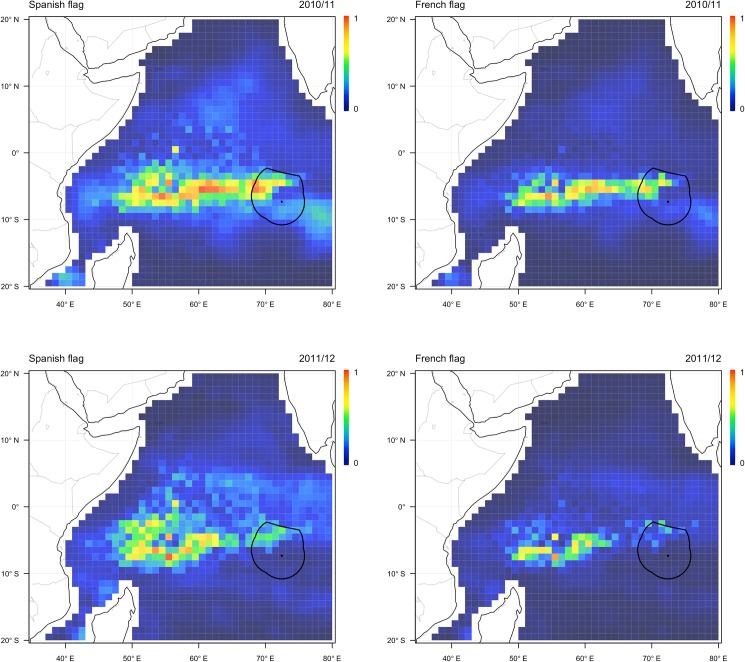
Predicted allocation of effort by the Spanish (left) and French (right) components of the fleet in the western Indian Ocean during the months December-January in 2010/2011 (upper) and 2011/2012 (lower). The location of the BIOT closure is shown by the solid line. Coloured cells show model predictions of probability of effort being allocated into an area.

In the first year of the designation of the BIOT reserve the fleet was expected to have fished across a large area of the western Indian Ocean, with core grounds predicted along a subequatorial band extending as far east as BIOT (Fig 3). These counterfactual predictions, which take into account the influence of environmental conditions on the allocation of effort, suggest that fishing conditions were favourable throughout BIOT and the surrounding region during this season. In the second year of the closure of BIOT the expected fishing grounds of the fleet were relatively constricted, particularly for the French fleet component. The predicted southern and eastern distribution of effort was markedly different to the previous year, with a substantially lower probability of effort being allocated within and to the southeast of BIOT. This contrast in the anticipated behaviour of fleet between the two years was most likely due to an anomalous shallowing of the Seychelles-Chagos Thermocline Ridge, characterised by a deeper-than-usual thermocline and reduced sea surface temperatures [17], which produced highly unfavourable fishing conditions throughout the BIOT region in the 2011/12 season.

**Fig 4:**
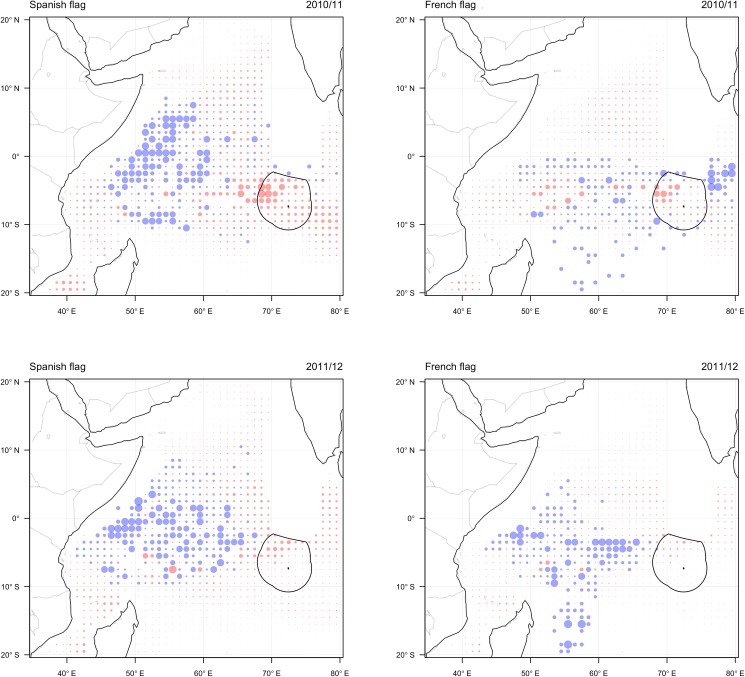
Observed allocation of effort by the Spanish (left) and French (right) components of the fleet in the western Indian Ocean during the months December-January in 2010/2011 (upper) and 2011/2012 (lower). Observations in each grid cell are shown as the predicted probability of effort minus observed response, where blue circles show more effort than expected and red circles show less effort than expected. Circle size indicates the relative size of the residual. The location of the BIOT closure is shown by the solid line.

In the observed scenario there was a marked difference in the allocation of effort by the French and Spanish components of the fleet in the first year of closure of BIOT. In the case of the Spanish fleet component, effort was mainly allocated in the Seychelles region as predicted, although some effort was allocated in the northwest Somali basin region (Fig 4). This suggest that a number of vessels remained in the main FAD fishing areas out of season. This is unexpected behaviour given the model predictions, although it is not possible to conclusively attribute it to the BIOT closure. The allocation of effort by the French fleet, whilst again mainly concentrated in the southwest Seychelles region, suggested considerable exploration around the BIOT reserve. A number of vessels travelled to the east of BIOT into areas rarely fished by the fleet, either passing through the closure or passing to the south. This fishing behaviour may be explained by poor fishing opportunities experienced elsewhere, prompting French skippers to search in novel areas around BIOT, or may reflect the behaviour of skippers attempting to assess the lost opportunities resulting from the designation of the reserve.

In the second year of closure of BIOT the fleet mainly allocated effort throughout the Seychelles region, with very little effort allocated in the proximity of the BIOT closure. This largely corresponded with predictions of the model and thus suggests little disruption of fishing behaviour caused by the reserve.

## Discussion

Retrospective analyses of the response of fishing fleets to closed areas are scarce, although from the handful of published examples a varied set of fleet responses has been demonstrated. For instance, [17] observed a concentration of effort by otter trawlers around the boundaries of closed areas in the northwest Atlantic, a phenomenon now widely termed ‘fishing the line’, and interpreted these patterns of effort reallocation as a response either to the spillover of biomass or the seasonal movement of fish out of the closures. A similar reallocation of effort was observed for the Atlantic tuna purse seine fleet around a temporary closed area, although given the short timescale considered this response was presumably linked to practical changes in where the fleet could operate rather than a response to increased tuna abundance [9]. In contrast, [18] found no evidence of vessels fishing the line in a Californian rockfish fishery, due either to the lack of a real or perceived spillover effect or an informal agreement between fishers and the reserve manager not to fish the boundaries. There is also some evidence to suggest that vessels may disperse widely when fishing grounds are closed in an attempt to escape the increased competition along closure boundaries [19,20].

In these examples, the reasons for the observed reallocation of effort around closed areas have generally been inferred and few studies have been able to disentangle the policy effect of a closure from other competing influences [10]. This is because most previous evaluations of closed area effects have used observation-based before-after approaches [6,7,9], which although able to identify changes in behaviour, cannot ascribe such changes completely to the closure of fishing grounds given the absence of a control site or other means of accounting for changes in the environmental and socio-economic drivers of fishing behaviour.

In this study we have demonstrated a counterfactual approach as a useful diagnostic tool in evaluating the performance of area closures. To our knowledge, this is the first use in the fisheries literature of a counterfactual approach for evaluation using model-based predictions, and therefore marks new territory. We did not focus on whether the closures achieved their stated fisheries and conservation objectives. Rather we focused on whether the policy of closing areas *per se* caused the fleet to reallocate fishing effort. The construction of a counterfactual scenario proved to be crucial in isolating the effect of the closures from confounding environmental influences on effort allocation and our results showed a mixed and inconsistent closure effect on fleet behaviour. Our results also demonstrated the flexibility of the fleet in adapting to the closures, particularly its ability to explore new areas, and furthermore we identified varied responses within the fleet depending on flag nationality, suggesting that the impact of closures may vary for different components of the fleet.

Of particular interest in our results was the inconsistent effect of the BIOT reserve on fleet behaviour. Comparison between the observed behaviour of the fleet and counterfactual predictions of effort allocation showed a response to the closure in the first year of its designation but not the second, most likely resulting from inter-annual differences in environmental conditions and hence the distribution of tuna schools. This result highlights the importance of placement of fixed closures in fishery systems characterised by high environmental variability where the complex suite of influences on fleet behaviour can make it difficult to predict or evaluate the contribution of a closure to achieving conservation and management objectives.

A second key result was the varying magnitude of closure effect on the two fleet components observed for both closures. In the case of the IOTC closure, the Spanish fleet component showed the most marked change in behaviour, with a number of vessels fishing to the east of the closure in typically un-fished areas, whereas the French fleet component allocated effort mainly in traditional grounds to the south of the closure. This varied response is largely explained by differences in the seasonal movement pattern of the fleet components linked to fishing strategy, with Spanish vessels typically remaining in the northwest FAD-fishing grounds for a longer period than French vessels, which move into subequatorial free schools regions earlier. A switch in the relative impact on two fleet components was observed for the BIOT closure in 2010/11, when the French component showed a more evident response to the closure with considerable exploratory behaviour around the closure boundaries. This variation in response is probably again linked to company-level strategy and differences in the targeting of free and associated schools, with Spanish skippers belonging to FAD-orientated companies less inclined to search for free schools in the vicinity of the BIOT closure and the opposite true for French skippers.

In this study we have examined the short-term response of the fleet to the two closed areas. It remains unclear how these or other similar closures might affect fishing opportunities over a longer period, including how any cumulative impact on catches in the long term might influence decisions at a company level to participate in the Indian Ocean fishery. Furthermore, we did not look at the overall effect of the IOTC and BIOT closures on catches of tunas or bycatch species and as such we cannot offer a conclusion as to the conservation efficacy of large offshore closures. The effect of closures on fish stocks can take years or decades to be demonstrated [21] and, as with fleet behaviour, it can be difficult to isolate the effect of a closure in generating reductions in catch or bycatch without accounting for other influences on production dynamics. The development of a model that integrates population dynamics of fishery-affected stocks with the model of fleet behaviour we employ here would be a valuable next step in the evaluation of the benefit of area closures for conservation and fisheries management.

## Supporting information

S1 Table Summary of the explanatory variables considered in the models, their predicted effect on effort allocation into an area and data sources. All variables were aggregated at monthly intervals and at a spatial resolution of 1° latitude/longitude.
(DOCX)
