# Supplementary material for: Use of a counterfactual approach to evaluate the effect of area closures on fishing location in a tropical tuna fishery
Source: PLoS One. 2017 Mar 29;12(3):e0174758. doi: 10.1371/journal.pone.0174758 (PMC5371335; doi:10.1371/journal.pone.0174758)
Supplement: S1 Table — All variables were aggregated at monthly intervals and at a spatial resolution of 1° latitude/longitude. (DOCX) [file pone.0174758.s001.docx]

Supplementary Information

## Appendix 1 Description of the model of fleet behaviour

Full details of the statistical fleet model are presented in [1]. Only a summary of the data used and modelling approach is presented here.

## Data

Purse seine fishing data were available from the Indian Ocean Tuna Commission (IOTC; www.iotc.org, downloaded Sept 2013). Data were disaggregated by flag nationality to distinguish between the French, including vessels flagged to French Territories (33.7% registered vessels), and Spanish, including Spanish-owned vessels flagged to Seychelles (53.1% registered vessels), components of the fleet as these had a consistent presence in the western Indian Ocean during the period analysed (2007-2012). The behaviour of the fleet was considered at the spatial resolution of 1° latitude/longitude grid cells and the temporal resolution of one month.

Data were obtained for three aspects of the biophysical ocean environment relevant to purse seine fishing; sea surface chlorophyll-*a* (SSC; mg/m^3^), sea surface temperature (SST; °C), and sea level anomaly (SLA; cm). All environmental variables were downloaded in 8-day intervals but averaged by month to correspond with fisheries data. Data for SSC and SST were obtained from measurements produced by the MODIS sensor, made available for download by the Distributed Active Archive Centre of the Goddard Space Flight Centre/NASA (available at http://disc.sci.gsfc.nasa.gov, downloaded August 2013). SSC was log transformed to improve the spread of skewed values. SLA data were obtained from information collected by the TOPEX and Poseidon altimeters, made available for download by Aviso (available at http://www.aviso.oceanobs.com, downloaded August 2013).

## Statistical modelling

A series of models was fitted to the data. The response variable was binary, indicating whether or not fishing effort was observed in a location in a given month. Eight explanatory variables were chosen to construct models, based on an understanding of the tools, techniques and fishing practices used by skippers to find tunas, although only variables that were relevant in explaining the behaviour of the fleet at a broad spatiotemporal scale considered (Table S1).

**Table S1.** Summary of the explanatory variables considered in the models, their predicted effect on effort allocation into an area and data sources. All variables were aggregated at monthly intervals and at a spatial resolution of 1° latitude/longitude.

| **Variable** | **Description** | **Range/units** | **Data source** |
| --- | --- | --- | --- |
| **Categorical variables** | | | |
| Year | Calendar year | 2007-2012 | - |
| Season | Quarterly period; February-April, May-July, August-October, November-January | 1-4 | - |
| Flag | Flag nationality of reported effort | France/Spain | IOTC |
| Past use | Frequency with which the location was fished in the same month in the previous five years by vessels of the same flag nationality | 0-5 | IOTC |
| **Continuous variables** | | | |
| SSC | Log-transformed sea surface chlorophyll-*a;* proxy for primary productivity | 0.02-25.8 mg/m^3^ | MODIS |
| SST | Sea surface temperature | 22-32 °C | MODIS |
| SLA | Sea level anomaly; proxy for thermocline depth | -36-50 cm | Topex/Poseidon |
| Distance | Distance from the port of Victoria, Seychelles (calculated using the Spherical Law of Cosines) | 0-3,000 km | - |

Four variables described the biophysical characteristics of the location; oceanographic conditions (*SST*, sea surface temperature; *SSC*, sea surface chlorophyll-*a* concentration; *SLA*, height of sea level anomaly) and meteorological conditions (*wind*, wind speed over the sea surface). The past behaviour of the fleet was described as the frequency that fishing effort reported by vessels of the same flag nationality was observed in the location in the same month in the previous five years, thus taking into account seasonality in the use of fishing grounds. The variable *distance* described the position of the location relative to the port of Victoria, which for simplicity was taken to be the main port used by the fleet. In addition to the main effects, the variables *year*, *month* and *flag* were included to account for possible temporal variation in the spatial footprint of the fleet.

## Autocorrelation

In this study there was a strong possibility of both temporal and spatial autocorrelation in the model residuals. Correlation plots were used to visually test for the presence of spatial and temporal autocorrelation in the residuals of a model fitted with all predictor variables. Autocorrelation function plots showed no significant temporal autocorrelation, and so no further action was taken. However, correlograms indicated moderate spatial autocorrelation to a lag distance of up to ~5 degrees. A number of approach have been described to deal with spatial autocorrelation in regression modelling, including the use of autocovariates or spatial eigenvector mapping [2], both of which were trialled in this study. However, these approaches introduced additional non-trivial issues that affected the fitting or interpretation of model results, and eventually a decision was made to proceed without attempting to address spatial autocorrelation.

## Model structure

A candidate set of generalised additive models (GAMs) was chosen *a priori* and fitted to the data using R 2.15 (R Development Core Team 2012) using the package *mgcv* [3]. Smooth functions were used to fit to the variables *SSC*, *SST*, *SLA, wind* and *distance*. Penalized cubic regression splines were used for computing efficiency due to the very large number of observation in the data. The Akaike information criterion (AIC) was used to rank and assign support for the competing candidate models. This selection criterion uses maximum likelihood scores as a measure of how well the model fits the data, taking into account model parsimony.

The data were split randomly into a training dataset (90%) and a validation dataset (10%), with the latter used to evaluate the predictive accuracy of the models using the area under the Receiver Operating Characteristic curve (AUC), where a score of 0.5 indicates that model accuracy is no better than random and a score of 1 indicates perfect discrimination [4].

## References

1. Davies TK, Mees CC, Milner-Gulland EJ. Modelling the spatial behaviour of a tropical tuna purse seine fleet. PloS One. 2014;9: e114037.

2. Dormann C, M. McPherson J, B. Araújo M, Bivand R, Bolliger J, Carl G, et al. Methods to account for spatial autocorrelation in the analysis of species distributional data: a review. Ecography. 2007;30: 609–628. doi:10.1111/j.2007.0906-7590.05171.x

3. Wood S. Generalized additive models: an introduction with R. CRC press; 2006.

4. Fawcett T. An introduction to ROC analysis. Pattern Recognit Lett. 2006;27: 861–874.
